# Draft Genome Sequences of Multidrug-Resistant Escherichia coli Strains Isolated from River Water in Malaysia

**DOI:** 10.1128/mra.00399-22

**Published:** 2022-06-09

**Authors:** Yus Amira Yusaimi, Nurul Syazwani Ahmad Sabri, Motoo Utsumi, Hirofumi Hara

**Affiliations:** a Graduate School of Life and Environmental Sciences, University of Tsukuba, Tsukuba, Ibaraki, Japan; b Institute of Biodiversity and Environmental Conservation, University of Malaysia Sarawak, Kota Samarahan, Sarawak, Malaysia; c Department of Chemical and Environmental Engineering, Malaysia-Japan International Institute of Technology, Universiti Teknologi Malaysia, Kuala Lumpur, Malaysia; d Microbiology Research Center for Sustainability, University of Tsukuba, Ibaraki, Japan; e Department of Biotechnology, Graduate School of Agricultural and Life Sciences, The University of Tokyo, Tokyo, Japan; University of Delaware

## Abstract

Antimicrobial resistance has become a primary concern in clinical and public health. Escherichia coli is one of the bacteria that carries and disseminates antimicrobial resistance genes to the community. Here, we report the draft genome sequence of three multidrug-resistant E. coli strains that were isolated from river water in Malaysia.

## ANNOUNCEMENT

Escherichia coli is a fecal coliform that contaminates aquatic environments from the discharge of human and animal waste, as well as from wastewater treatment plants (1–3). Selective pressure from the environment may cause these bacteria to acquire resistance genes and serve as a significant reservoir for the transmission of antimicrobial resistance (AMR) determinants, becoming a threat to global health (4–6). Here, we sequenced three E. coli strains that were isolated from river water as reported previously (7) to determine their responsible AMR determinants. River water receiving treated and untreated wastewater discharged from nearby hospitals and medical service centers was collected from the Gombak River (3°10′13.2″N, 101°41′41.6″E) in Kuala Lumpur, Malaysia. Two chromogenic selective media, that is, Chromocult coliform agar (Merck Millipore) and eosin methylene blue agar (Nissui Pharmaceutical Co., Ltd.), were used to isolate the presumptive E. coli strains (8, 9), with further confirmation by the detection of *uidA* and *uspA* genes using PCR. The multidrug-resistant (MDR) strains were then obtained by disk diffusion assay (10). The MDR E. coli strains GR2, GR3, and GR5 (7) were selected for whole-genome sequencing. Genomic DNA was extracted from overnight cultures grown on Luria-Bertani (LB) plates using an ISOIL for Beads Beating kit (Nippon Gene) following the manufacturer’s protocol, with modification of the centrifugation speed and time to 12,000 rpm for 10 min. Paired-end libraries were prepared using a MGIEasy FS DNA library preparation set (MGI Tech Co., Ltd.) according to the manufacturer’s protocol. The libraries were quantified using a DNF-915 double-stranded DNA (dsDNA) 915 reagent kit and Fragment Analyzer automated CE system (Advanced Analytical Technologies, Inc.), followed by sequencing using the DNBSEQ-G400RS high-throughput sequencing set (Bioengineering Laboratory, MGI Tech Co., Ltd.), generating 2 × 200-bp sequence reads. Raw sequencing data were trimmed and filtered using CLC Genomics Workbench v11.0.1 to obtain clean reads. Sequencing adapters were trimmed from the raw sequences, and the sequences were merged into paired reads. The paired reads were trimmed with the parameters of quality score limit of 0.05, discarded reads of <400 nucleotides, and a maximum number of ambiguous nucleotides, followed by assembly with the default parameters. Assembly metrics were then evaluated using QUAST v5.0.2 (11) and annotation with NCBI PGAP v5.1 (12) (Table 1).

**TABLE 1:** Genomic features of Escherichia coli strains GR2, GR3, and GR5

Strain	Total no. of reads	Sequencing depth (×)	No. of reads	Genome size (bp)	No. of contigs	*N*_50_ (bp)	G+C content (%)	No. of CDSs^*a*^	No. of RNAs	No. of tRNAs	SRA accession no.
GR2	1,758,541,600	160	4,396,354	4,882,886	114	139,964	50.5	4,521	11	75	SRR16588251
GR3	1,695,898,000	154	4,239,745	5,198,255	149	109,874	50.5	4,833	13	75	SRR16588250
GR5	1,857,770,000	169	4,644,425	5,460,747	204	100,204	50.6	5,168	6	75	SRR16588249

a CDS, coding DNA sequence.

Analysis by AMRFinder v3.9.8 (13) revealed the presence of multiple antibiotic genes that confer resistance to β-lactams (*bla*_TEM-1_, *bla*_TEM-176_, *bla*_CTX-M-65_, and *bla*_CMY-2_), tetracyclines (*tetA*, *tetX*, and *tetM*), aminoglycosides [*aac(3)-Iva*, *aac(3)-IIe*, *aadA1*, *aadA2*, *aphy(3′)-Ib*, *aph(3″)-Ib*, *aph(4)-Ia*, and *aph(6)-Id*], sulfonamides (*sul2* and *sul3*), phenicols (*florR* and *cmlA1*), quinolones (*qnrS1*), trimethoprim (*dfrA1*, *dfrA2*, and *dfrA14*), and fosfomycin (*fosA3* and *fosA4*) (Table 1). In addition, genes encoding the major facilitator superfamily (MFS), resistance-nodulation-cell division (RND), and ATP-binding cassette (ABC) antibiotic families of efflux pumps were discovered by CARD (14) analysis. The genome information on E. coli strains depicts the high prevalence of resistance genes in tropical aquatic systems. Hence, diligent surveillance of MDR strains in the rapidly changing aquatic environment will enable continued surveillance of resistance in the tropics.

### Data availability.

The raw data for all Escherichia species strains were deposited in GenBank under BioProject accession number PRJNA756839 and BioSample accession numbers SAMN20951119 (GR2), SAMN20951120 (GR3), and SAMN20951121 (GR5). GenBank accession numbers for strains GR2, GR3, and GR5 are JAKNRN000000000, JAKNRO000000000, and JAKNRP000000000, respectively. The SRA numbers are provided in Table 1.
